# Case of Obstinate Plethora, with Violent Hæmorrhagic Oscillations in the Balance of the Circulation

**Published:** 1818-07-01

**Authors:** Mathias Felix

**Affiliations:** Bristol


### *Case of obstinate Plethora, with violent Hæmorrhagic Oscillations in the Balance of the Circulation.*
### By Mathias Felix, M. D. of Bristol.

Augustus Rogere, a French prisoner of war at Stapleton, 38 years of age, was brought into the hospital on the 9th of October, 1807, in a state of syncope, occasioned by a profuse hæmorrhage from the anus. The appearance of the blanket and hammock in which he was carried, and the coagula therein, shewed that he must have lost at least five or six pounds of blood! The hæmorrhage had ceased on the supervention of syncope, but returned with the animation and warmth, attended with a full hard pulse. A pound of blood was abstracted from the arm, and glysters of cold water were thrown up: infusion of roses with sulphuric acid, was given in large quantities, together with cold acidulated drinks. The hæmorrhage was again checked; and the bowels were opened by means of castor oil.

11 *Oct.*—Complains of great oppression and pain in the chest, with full, hard pulse. Venesection to sixteen ounces; a large blister to the breast, and another to the back; saline cathartic mixture. By these means the thoracic symptoms were relieved, and he continued better till the 19th, when he complained of great pain and tension of the abdomen. Emollient glysters; castor oil; the warm bath; leeches to the abdomen, and afterwards a blister. These brought relief; and small doses of calomel were given every night, with an opening saline draught occasionally.

1*st. Nov.*—The pain in the chest has returned, with cough and dyspnœa. Venesection to sixteen ounces; a mixture with digitalis. 2*d.*—Worse. Oppression very great; vessels of the face and neck turgid. Venesectio ad. ℥xx. The oppression, &c. continuing, twenty ounces more were abstracted; calomel and antimonial powder, of each four grains, every five hours, with a saline mixture, and a blister to the breast. 3*rd.*—Bled again to sixteen ounces, and nitre added to the mixture: bowels open. 4*th.*—Hard pulse; difficult respiration, with pain; violent cough; venesectio ad. ℥xvj. Another venesection of ℥xvj. in the afternoon. 5*th.*—Breathing less difficult; cough hard; bowels open. *Contin. Medicamenta.*

7*th.*—Complains of weakness, but says he feels the blood coming again to his breast. Pulse full; venesectio ad. ℥xij. 8*th.*—Repet. venesectio, mistura cathartica *pro re nata.* 12*th.*—His face is again turgid; pulse hard; breathing difficult and painful. Leeches to the chest; but not affording relief, he was bled to twenty-four ounces, and a blister applied to each side. It may here be observed, that the blood uniformly exhibited the true buffy coat of pulmonic inflammation. At first he considered the Sangrado practice as truly barbarous; but latterly he experienced so much benefit from the measure, that he was always anxious to have venesection performed

From some idea that the liver was in fault, I directed a drachm of mercurial ointment to be rubbed in, for three or four nights; but as he appeared to get worse, it was discontinued.

26*th.*—The difficulty of breathing has returned, with acute pain in the side, and troublesome cough. He now appears the emblem of debility. He is pale; emaciated; the countenance sunk; the eyes hollow; the appetite entirely gone. It therefore became a serious question what was to be done! I had not found that benefit from *local* bleeding, purging, blisters, and digitalis, &c. which I expected; but general bleeding was certain relief, and to *bleed or die* seemed now the only alternatives. There was a morbid disposition to be conquered, else it must conquer us: accordingly I took away this day *forty-four ounces* of blood, and prescribed from ten to fifteen drops of tincture of digitalis every six hours. Things seemed better; but next day (27) I found it necessary to abstract twenty more ounces. We at last appeared to have subdued the enemy; the pulse kept quiet, and the chest was easy; small blisters, however, were applied to the thoracic parietes.

*December 17th.* The hæmorrhoids are become troublesome, and leeches have been applied to them; the flow of blood was long continued and free; the relief considerable. On the 23d, the old symptoms returned, but in a slighter degree; and gave way to a venesection of twelve ounces. On the 24th, it was necessary to repeat the bleeding, and again on the 31st. From this time we had no active disease to resist. The blood also, in the last venesection, appeared to have lost its inflammatory qualities. But digitalis was long continued, with purgatives, and the most rigid abstinence in respect to diet. A seton was established in the side, with the view of taking off the determination to internal organs, and of keeping up a permanent drain from the system. On the 2d of March he was discharged from the hospital, cured.

In respect to his previous history, he had been in hospital, about eighteen months previously, for a violent pulmonary hæmorrhage; and had then lost a vast quantity of blood, both from the lungs and the arm; and after the usual routine treatment, he had been discharged into the prison, where he had been employed by the masons then building an extensive boundary wall, as a labourer to make and carry mortar, lift heavy stones, &c. which he continued to do, without any complaint, for eight or ten months. To conclude, he allowed his seton to heal up soon after he went out of hospital, and became strong and robust as ever, and never afterwards suffered any kind of illness, during three years that he remained in prison.

On an examination for invaliding, Rogere presented himself as an object for discharge from captivity. As he was the picture of rude health, he was asked, with no small degree of surprise, by the Inspector, what was the matter with him? He answered, "Nothing;" but that he conceived he had a fair claim for release after being "four or five months in the hospital, where he had lost as much blood, and swallowed as much physic, as would swim a frigate, besides having nearly all the skin of his body taken off by blisters, &c."! This affecting tale of poor Rogere, which was not much exaggerated, after due allowance for French amplification, had not the desired effect on the examiners, who remanded him to his prison, from whence he was only released by a general peace.

This case, to which I scarce know how to give a name, was remarkable for the proneness to irregular vascular action, and sanguification, notwithstanding the numerous outlets which were given by bleeding, purging, &c. while the rigid regimen seemed to preclude the possibility of fresh supplies. Yet the blood was always richly buffed, and firmly cupped; frequently of a florid arterial colour. These appearances continued till the last; in short, till the corporeal fabric was reduced almost to bone and skin, and till there was no longer a supply for the vascular system through the medium of the absorbents. I think it probable, that in this case, we only restrained, by artificial means, the morbid action from overpowering some organ essential to life, till nature and art together brought the whole body to that state which furnished no farther materials for this morbid process. It is not improbable, that it is *in this way*, we prove nature's handmaids on many other occasions, when we think we have been the sole operators ourselves. At all events, the above case may, I think, not be unworthy of notice in these phlebotomising days, when the timidity of *former* practice is in every body's mouth.

*Bristol, January,* 1818.

The Editor returns many thanks to Dr. Felix, for the above very interesting case, which certainly does credit to the Stapleton practice, at a time when lancets were in *comparatively* small demand.

